# Generative Adversarial Network-Based Joint Mapping and Localization for Millimeter-Wave Communication Systems

**DOI:** 10.3390/s26134319

**Published:** 2026-07-07

**Authors:** Zexu Zhao, Zhigang Chen, Lu Chen

**Affiliations:** School of Information and Communications Engineering, Xi’an Jiaotong University, Xi’an 710049, China

**Keywords:** angle difference of arrival (ADOA), deep neural network, generative adversarial network (GAN), joint localization and mapping (JLAM), millimeter-wave communication systems

## Abstract

In this paper, we propose a novel generative adversarial network (GAN)-based joint localization and mapping (JLAM) method using angle difference of arrival (ADOA) measurements for millimeter-wave (mmWave) communication systems. The proposed method adopts a deep auto-encoder neural network as the discriminator of the GAN and models the generator as an explicit geometric ADOA function of the access point (AP) positions and the mobile terminal (MT) position, rather than as a conventional black-box neural network. By exploiting the two-dimensional distribution characteristics of high-dimensional ADOA vectors collected at a large number of random and unknown MT positions, the proposed method learns the ADOA data distribution and transforms it into the AP geometric topology. Then, the MT positions and the indoor map are estimated based on the recovered physical and virtual AP topology. The simulation results show that, under the representative setting with N=2000 measured ADOA vectors and σ=2° AOA measurement noise, the proposed method achieves an average localization error of about 0.25 m, compared with about 0.60 m for the JADE algorithm, corresponding to an error reduction of approximately 58%. The proposed method also provides more accurate room boundary estimation than JADE, confirming its effectiveness for mmWave JLAM.

## 1. Introduction

Indoor wireless localization has become an important research topic in recent years, driven by its broad potential applications in areas such as smart construction, healthcare services, pedestrian navigation, and indoor guidance. Existing indoor positioning techniques are generally categorized into two representative groups: geometry-based localization methods and fingerprint-based localization methods [1]. The geometrical location methods usually employ the multipath parameters, such as time-of-arrival (TOA) or angle-of-arrival (AOA), to realize positioning through geometric principles [2,3]. Due to the serious non-line-of-sight (NLOS) multipath effects and the limited system bandwidth, such geometric location methods suffer from limited estimation accuracy of multipath parameters [2,3,4]. In contrast, fingerprinting-based localization usually determines the target position by matching real-time RSS or CSI measurements with an offline radio map constructed in advance. Typical examples include nearest-neighbor (NN) and K-nearest-neighbor (KNN)-based methods [1,5]. Since the measured fingerprints implicitly contain the propagation characteristics of both LOS and NLOS components, such methods are able to exploit location-dependent information embedded in multipath signals. However, the multipath effects make the received signal strength (RSS) or channel impulse response (CIR) fingerprints spatially unstable, leading to the unstable localization performance. Even worse, the fingerprinting positioning methods require a prohibitively huge workload for constructing an offline fingerprint database [5]. Hence, the conventional positioning methods, including the geometric positioning methods and fingerprinting methods, are subject to multipath effects.

Millimeter-wave (mmWave) communication has been widely regarded as one of the enabling technologies for supporting multi-Gbit/s data transmission in 5G and future wireless networks [6,7]. Owing to the large available bandwidth and the compact antenna size associated with millimeter wavelengths, mmWave systems can provide fine resolution in both the delay and angular domains [8,9]. In addition, mmWave signals generally exhibit quasi-optical propagation behavior and sparse multipath characteristics, mainly because of their weak scattering capability in typical indoor environments [10,11]. To sum up, the mmWave communication systems can provide great potential for achieving high positioning accuaracy due to the multipath sparsity and high temporal and spatial resolution of mmWave channels, which is not the case for sub-6GHz microwave propagation and means more information can be extracted from the multipath parameters [12]. As a result, lots of mmWave positioning methods through processing RF multipath measurements have been proposed to enhance the positioning accuracy [13].

However, the existing multipath measurement processing-based mmWave positioning methods are severely subject to extra requirements for deterministic or statistical information beyond mmWave communications for overcoming the problem of unknown virtual AP (access point) positions, which are the reflection mirrors of the physical AP through indoor plane surfaces. On the one hand, fingerprint-based mmW positioning methods can make full use of multipath information without estimating multipath parameters and thus can achieve better performance [6,14,15,16,17], whereas this category of methods requires laborious and complex offline database work. On the other hand, mmW multipath parameter-based geometrical positioning methods can improve the positioning accuracy by using the extra geometric relationship between indirect path measurements and the mobile terminal (MT) position, while these methods still suffer from the rigorous assumptions or requirements on the indirect path parameters due to the unknown complex environments. For example, refs. [9,18,19,20] exploit both the direct path angle and indirect path angles to realize location by strictly assuming information of indirect path reflectors or the known floorplan, and [21,22] estimate the MT position by geometrically resolving the information redundancy provided by a parameter triplet of each path, TOA, AOA and AOD (angle-of-departure), which require not only extra-strict synchronization between mmWave transmitters and receivers but also the known bearings of antenna arrays at the AP and/or the MT.

As a viable solution to the problem of virtual APs’ unknown positions for practical mmW communication systems, a few zero-initial-information-based mmW joint localization and mapping (JLAM) methods have been proposed to estimate AP locations and MT locations jointly, these methods usually gather information of multipath parameters at a large number of positions and then employ the information redundancy of such multipath measurements to localize the MT as well as the APs [9,19]. Since the angle difference of arrival (ADOA) can be obtained from the beam-training results already available in practical mmWave devices, several ADOA-based zero-initial-information JLAM methods have been developed. For example, the Joint Anchor and Device location Estimation (JADE) algorithm in [9] reformulates the originally NP-hard joint estimation problem of AP coordinates and random MT positions into an iterative procedure consisting of two alternating least-squares (LS) estimations. One LS step updates the AP positions, while the other estimates the MT positions. However, this simplification is achieved by relaxing the original geometric consistency among the AP topology, MT locations, and ADOA observations, which inevitably discards part of the information carried by the ADOA measurements. In addition, the communication-driven localization and mapping (CLAM) method in [19] first recovers the physical and virtual AP positions by solving a minimum set of equations that relate the ADOA observations to the AP shape, where these equations are obtained offline through automatic symbolic manipulation. The MT position is then inferred using a robust ADOA localization procedure. Nevertheless, in order to keep the computational burden manageable, CLAM does not exploit all the available interdependencies among the ADOA measurements, which may also lead to a degradation in estimation performance. Therefore, the existing zero-initial-information-based mmW SLAM methods have to compromise between low complexity and high information utilization since a large number of ADOA measurements are gathered to accumulate enough information.

In this paper, a novel generative adversarial network (GAN)-based mmWave JLAM algorithm has been proposed. Without requiring any extra information beyond the capability of MMW communication devices, the proposed algorithm first employs a deep neural network-based discriminator in the GAN to learn the 2-dimensional statistical characteristics of crowd-sourcing or online multipath ADOA vectors measured at a large number of random and unknown positions, which is completely dependent on the AP geometric topology. Further, it can transform such extracted 2-dimensional statistical characteristics into the AP geometric topology or AP positions through optimizing the generator in the GAN, since the generator is explicitly modeled as the ADOA function of the AP topology and the MT position, rather than a deep neural network. Based on the estimated AP positions, the MT position corresponding to each ADOA vector can be estimated through classical triangulation location schemes and the room boundary can be geometrically calculated as the reflection points, i.e., the environment map is constructed. Considering the GAN can take more advantage of the geometrical information of the crowd-sourcing and/or online ADOA data, our GAN-based SLAM method can achieve high information utilization, thus can outperform the existing mmW JLAM methods. This work extends our previous research on mmWave SLAM for joint positioning and mapping [23]. Our previous study introduced an Expectation Maximization (EM)-based SLAM method for estimating AP and MT positions. However, this EM based approach is highly sensitive to initial parameter settings and relies on the assumption of Gaussian-distributed data. In contrast, this paper introduces a novel GAN-based JLAM algorithm, which learns the 2D distribution of high-dimensional ADOA vectors through adversarial training, enabling improved robustness and generalization without strong distributional assumptions.

Different from existing GAN-based localization methods that mainly use GANs for fingerprint generation, data augmentation, or semi-supervised location regression [24], the proposed method constructs the generator as an explicit geometric ADOA function. Therefore, the GAN is used to estimate the physical and virtual AP topology from unlabeled ADOA vectors, rather than to directly generate fingerprints or regress MT positions.

The rest of this paper is organized as follows. In Section 2, the virtual AP-based system model is described and the multipath ADOA vector is defined. We develop a novel ADOA and GAN-based SLAM method, mainly including the AP topology estimation by training the GAN and positioning MTs through classical schemes, and analyze the Cramer–Rao lower bound for positioning the MT in Section 3. The simulation results are presented in Section 4 to evaluate the performance of the proposed method. Finally, conclusions are drawn in Section 5.

## 2. System Model

**Figure 1:**
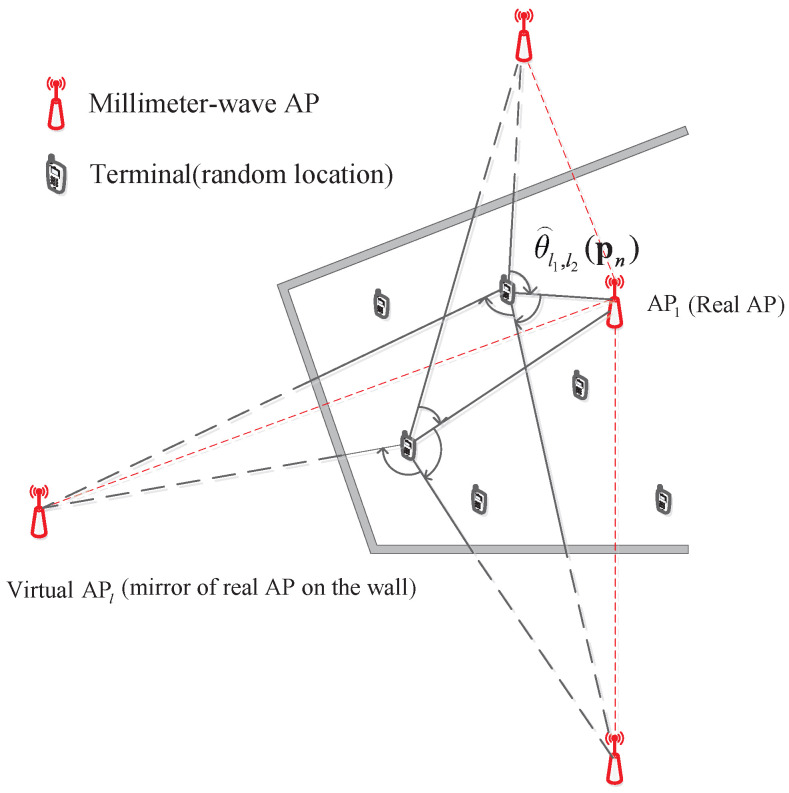
Virtual AP-based mmW system model.

Consider a 2-dimensional indoor scenario (shown in Figure 1) in which a single mmWave AP, denoted as ${AP}_{1}$, is deployed and its location $a_{1}$ is unknown. The following system model can be simply extended to a 3-dimensional scenario. Because mmWave signals suffer from severe propagation loss and approximately follow optical-like propagation in air [10], this work considers only the LOS path and the single-bounce reflected paths, following the modeling assumptions in [9,19]. Reflected paths involving two or more bounces are assumed to be negligible, since they usually undergo much stronger attenuation. A first-order NLOS component can be equivalently represented as an LOS component transmitted from a virtual AP, which is the mirror image of the physical AP with respect to the corresponding reflecting surface, such as an indoor wall. Let these virtual APs be denoted by ${AP}_{l}\left(l=2,3,\cdots ,L\right)$, with unknown coordinates $a_{l}={\left[a_{l,x},a_{l,y}\right]}^{T},\left(l=2,3,\cdots ,L\right)$. The complete AP set, including both the physical AP and the virtual APs, is then written as $A=\{AP_{1},AP_{2},\cdots ,AP_{L}\}$, where *L* denotes the total number of APs.

When the MT is located at $p_{n}\left(n=1,2,\cdots ,N\right)$, it can utilize the beam-training results naturally provided by the mmWave communication procedure to extract the AOAs of dominant multipath components. The ADOA measurements are then obtained by calculating the angular differences between the paths associated with different AP pairs, where the APs may correspond to either the physical AP or the virtual APs. In this work, the AOA/ADOA measurement vectors are used as the input of the proposed GAN-based JLAM method and are assumed to have already been obtained from the mmWave communication process, such as beam training or channel estimation. Therefore, this paper focuses on the localization and mapping algorithm based on the collected ADOA measurements, rather than on the detailed antenna-array design or the specific AOA estimation procedure. Denote the AOA of the signal from $AP_{l}$ to the MT located at $p_{n}={\left[p_{n,x},p_{n,y}\right]}^{T}$ as $\theta_{l}\left(p_{n}\right)$, thus the ADOA for the AP pair $\left\{AP_{l_{1}},AP_{l_{2}}\right\}$ at position $p_{n}$ can be expressed as ${\overset{˘}{\theta }}_{l_{1},l_{2}}\left(p_{n}\right)=\theta_{l_{1}}\left(p_{n}\right)-\theta_{l_{2}}\left(p_{n}\right)$. According to the properties of analytic geometry, the ADOA ${\overset{˘}{\theta }}_{l_{1},l_{2}}\left(p_{n}\right)$ satisfies

(1) $$\cos{\overset{˘}{\theta }}_{l_{1},l_{2}}\left(p_{n}\right))=\frac{\left(a_{l_{1}}-p_{n}\right)•\left(a_{l_{2}}-p_{n}\right)}{\left|\left(a_{l_{1}}-p_{n}\right)\right|\cdot \left|\left(a_{l_{2}}-p_{n}\right)\right|}$$

where • denotes dot-product operation.

Based on the observed ADOAs for different AP pairs, we construct the measured ADOA vector at the position $p_{n}$ as $\psi \left(p_{n}\right)=\left[{\hat{\theta }}_{1,2}\left(p_{n}\right),{\hat{\theta }}_{2,3}\left(p_{n}\right),\cdots ,{\hat{\theta }}_{L-1,L}\left(p_{n}\right)\right]$, where ${\left\{{\hat{\theta }}_{l-1,l}\left(p_{n}\right)\right\}}_{l=1}^{L-1}$ are the observed ADOAs. Further, we collect the ADOA observation vectors at a larger number of random and unknown positions and set up a dataset of ADOA vectors as $Q={\left\{\psi \left(p_{n}\right)\right\}}_{n=1}^{N}$. The preliminary ADOA measurement database is acquired as follows. When an MT appears at a random and unknown position, it performs the standard mmWave beam-training or beam-refinement procedure to identify the dominant LOS/NLOS paths and estimate their AOAs. The ADOA values are then obtained by subtracting the AOAs of different path/AP pairs, and the corresponding ADOA vector is stored as one unlabeled sample in Q. Repeating this process for many random MT positions, either through crowd-sourcing users or online measurements during normal communication, produces the ADOA dataset used for GAN training. Different from RSSI fingerprinting, the proposed ADOA database does not require pre-surveyed reference-point coordinates or location-labeled RSSI fingerprints; it only requires unlabeled ADOA vectors measured at random positions.

As indicated by (1), the constructed ADOA vector depends only on the relative geometry between the MT and the APs, and is therefore not affected by the unknown orientation offset of the MT. In typical mmWave multipath scenarios, the number of dominant paths, or equivalently the number of physical and virtual APs *L*, is usually greater than three. Consequently, if the AP positions are given, the ADOA vector contains redundant angular constraints for determining a two-dimensional MT position. When ADOA vectors are collected from many different and unknown MT locations, this redundancy provides sufficient geometric information to jointly infer the AP topology and the corresponding MT positions, including both the physical AP and the virtual APs.

Therefore, the problem of mmWave simultaneous localization and mapping for a mmWave communication system in an unknown indoor environment can be described as *simultaneously estimating the geometric topology of APs (including both physical APs and virtual APs) and the MT location based on the measured ADOA data $Q={\left\{\psi \left(p_{n}\right)\right\}}_{n=1}^{N}$, i.e., a large number of collected ADOA vectors at unknown MT locations.*

## 3. Algorithm

In this section, a novel generative adversarial network (GAN)-based AP topology estimation method is first proposed. Then, the structure and training method of the discriminator and generator of the GAN is described in detail. Further, based on the estimated AP topology and the measured ADOA vectors, a least-square positioning method for the MT is developed and the environment map is geometrically calculated as the reflection points.

### 3.1. GAN-Based AP Topology Estimation

For a given indoor environment or a given AP topology, the set of high-dimensional ADOA vectors observed at a large number of random MT locations can be regarded as a two-dimensional manifold inside a space of L−1 dimension, since an L−1 dimensional ADOA vector is mapped from a two-dimensional position coordinate. In other words, the dataset of ADOA vectors possess two-dimensional distribution characteristics. Such two-dimensional distribution characteristics are completely determined by the AP topology. Hence, the estimation of AP topology based on the collected ADOA dataset can be realized through learning the low-dimensional distribution characteristics of the ADOA dataset and transforming the low-dimensional distribution characteristic into the AP shape.

It should be noted that the two-dimensional characteristic mentioned here refers to the intrinsic manifold structure of the ADOA dataset, rather than a strict lossless reduction of all high-dimensional information to two independent variables. For a fixed AP topology, each ADOA vector is generated by a nonlinear geometric mapping from the two-dimensional MT position. Therefore, although the ADOA vector lies in an L−1-dimensional measurement space, its dominant intrinsic degrees of freedom are mainly determined by the two-dimensional MT coordinate. The higher-order dependencies among different ADOA components are still contained in the high-dimensional ADOA vector and are used by the discriminator during reconstruction and adversarial classification.

As an unsupervised learning-based generative network [25,26], the typical GAN including two neural networks (generator and discriminator) can learn low-dimensional features from a large number of ADOA data samples and then generates data with the same distribution as the original data. By using such unsupervised learning capability of the GAN, we present a GAN-based AP topology estimation method, which jointly learns the low-dimensional distribution characteristics of the unlabeled ADOA vectors and transforms it into the AP shape. The structure of the proposed GAN is shown in Figure 1.

The proposed GAN is composed of two parts: the generator that tries to generate the ADOA vector as real as possible and the discriminator that distinguishes the real ADOA vectors from the generated ones. As in typical GANs, our discriminator is implemented by a deep neural network, which first learns the low-dimensional characteristics from the real but unlabeled high-dimensional ADOA vectors, and then distinguishes the real vector from the generated vector. Different from the common generative models in GANs, our generator is realized explicitly based on the geometric relationship among the AP topology, the random indoor position and the ADOA vector in (1), rather than using a deep neural network. Hence, the generator’s parameters are the AP topology parameters. It should be emphasized that the generator in the proposed GAN is not a neural network-based black-box mapping. For a given random MT position, the generator is a deterministic and explicit geometric function derived from the ADOA relationship. The only trainable parameters of this function are the coordinates of the physical AP and virtual APs. Therefore, the adversarial training of the generator is actually a parameter estimation process for the AP coordinates. In other words, “transforming the low-dimensional distribution characteristics into the AP shape” means adjusting the AP-coordinate parameters in the explicit ADOA generator until the generated ADOA vectors have the same distribution as the measured unlabeled ADOA vectors.

As shown in Figure 1, with the input of randomly drawn 2-dimension positions of the MT, the generator generates fake ADOA vectors according to the ADOA function in (1). Further, the discriminator receives both the generated ADOA vector and the real ADOA vector and aims to tell them apart. Since the error signal to the discriminator is provided through the simple ground truth of knowing whether the ADOA vector came from the real measurements or from the generator, the error signal can be used to train both the generator and the discriminator simultaneously, leading the generator and the discriminator toward being able to produce forgeries of better quality and distinguish the real vectors from the generated vectors, respectively. Through the iterative training of the discriminator and the generator, the GAN can create ADOA vectors following the same two-dimensional distribution of the real ADOA vectors, thus the optimal estimation of AP coordinates is obtained.

The training of the discriminator and the generator is respectively developed in detail as follows. Without losing generality, the AP topology is represented as the AP coordinates with two AP coordinates fixed in the following training, since an AP topology has the ambiguity of rotation, translation and scaling.

#### 3.1.1. Training the Discriminator

**Figure 2:**
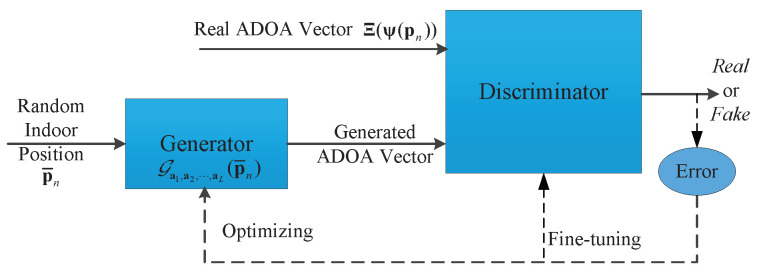
The GAN system for AP topology estimation.

To learn the two-dimensional feature of high-dimensional measured ADOA vectors, the discriminator in the proposed GAN system is modeled as a deep auto-encoder neural network, which is shown in Figure 2, and the Sigmoid function is adopted as the activation function of the hidden layers and the logistic regression layer.

In order to simplify the explicit function of the generator and ensure the data consistency between the generated samples and the training samples for the discriminator, we define the training sample of the discriminator as the following vector based on the ADOA measurements: $\Xi \left(\psi \left(p_{n}\right)\right)=\left[\cos\left({\hat{\theta }}_{1,2}\left(p_{n}\right)\right),\cos\left({\hat{\theta }}_{2,3}\left(p_{n}\right)\right),\cdots ,\cos\left({\hat{\theta }}_{L-1,L}\left(p_{n}\right)\right)\right]$.

##### Auto-Encoder Neural Network

As an unsupervised learning network, the auto-encoder neural network can automatically extract features from unlabeled data and give better feature description than original data. Assume that the auto-encoder neural network includes one input layer and M−1 hidden layers. Denote the parameters of *m*-th layer neural network as $\left(\omega^{\left(m\right)},b^{\left(m\right)}\right)$, where $\omega_{i,j}^{\left(m\right)}$ denotes the weight between the *i*-th neuron of the *m*-th layer and the *j*-th neuron of the (m+1)-th layer, and $b_{j}^{\left(m\right)}$ denotes the bias of the *j*-th neuron of the (m+1)-th layer. Denote the input of the *m*-th layer of auto-encoder neural network as ${\tilde{X}}^{\left(m\right)}\left(n\right)$, the input ${\tilde{X}}^{\left(m+1\right)}\left(n\right)$ and the decoding output $Y^{\left(m+1\right)}\left(n\right)$ of the (m+1)-th layer can be expressed as

(2) $${\tilde{X}}^{\left(m+1\right)}\left(n\right)=F\left(\omega^{\left(m\right)}{\tilde{X}}^{\left(m\right)}\left(n\right)+b^{\left(m\right)}\right)$$

and

(3) $$Y^{\left(m+1\right)}\left(n\right)=F\left({\left(\omega^{\left(m\right)}\right)}^{T}{\tilde{X}}^{\left(m+1\right)}\left(n\right)+{\overset{´}{b}}^{\left(m\right)}\right),$$

respectively, wherein ${\overset{´}{b}}^{\left(m\right)}$ represents the bias item of the corresponding deconding layer and F(•) represents the activation function, i.e., the Sigmoid function

$$F\left(z\right)=\frac{1}{1+exp(-z)}$$

Given the ADOA dataset, the auto-encoder neural network can be trained without supervision through minimizing the following loss function:

(4) $$\begin{array}{c} J\left(\omega^{\left(m\right)},b^{\left(m\right)},{\overset{´}{b}}^{\left(m\right)}\right)=\frac{1}{N}\sum_{n=1}^{N}{∥Y^{\left(m+1\right)}\left(n\right)-{\tilde{X}}^{\left(m\right)}\left(n\right)∥}^{2}\\ +\lambda \sum_{i}\sum_{j}{∥\omega_{ij}^{\left(m\right)}∥}^{2}+\gamma \sum_{n=1}^{N}\left|{\tilde{X}}^{\left(m+1\right)}\left(n\right)\right| \end{array}$$

where the first term denotes the average of the square reconstruction error, the second term and the third term are added to prevent the neural network from overfitting and to impose a sparsity constraint on the outputs of the neurons, respectively. Correspondingly, λ and γ denote the weight decay coefficient and the sparsity parameter, respectively.

The sparsity constraint in the auto-encoder is used to reduce overfitting to sample-specific noise in the spatial angle dataset. Let denote the average activation of the -th hidden neuron, and let denote the desired sparsity level. The sparsity penalty can be written as

(5) $$\Omega_{\mathrm{sparse}}=\sum_{j}KL\left(\rho ∥{\hat{\rho }}_{j}\right)=\sum_{j}\left[\rho \log\frac{\rho }{{\hat{\rho }}_{j}}+\left(1-\rho \right)\log\frac{1-\rho }{1-{\hat{\rho }}_{j}}\right]$$

This penalty encourages only a small subset of hidden neurons to be activated for each ADOA vector, so that the discriminator learns stable manifold features rather than memorizing noise in the measured spatial angles. Standard dropout is not adopted because ADOA vectors are structured geometric observations, and randomly dropping hidden units may disturb the geometric continuity of the learned ADOA manifold. In contrast, sparsity regularization preserves deterministic feature propagation while controlling the model capacity.

The input of the discriminator includes the real ADOA vectors and the generated vectors, which are respectively labeled as true or false. As the features of the real vectors and the generated vectors are extracted automatically by training the auto-encoder network, the probability of being true or false can be further judged by a follow-up logistic regression network.

##### Logistic Regression Network

**Figure 3:**
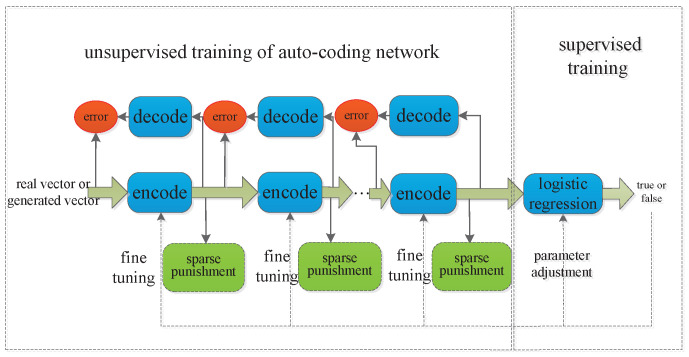
Auto-encoder neural network structure of the discriminator.

As shown in Figure 3, the output of a multilayer auto-encoder neural network ${\tilde{X}}^{\left(M\right)}\left(n\right)$ is input to a logistic regression network with a weight vector $\bar{\omega }$. The sigmoid function is also adopted as the activation function in this logistic regression network. Thus the output of logistic regression network can be written as

(6) $$D_{\bar{\omega }}\left({\tilde{X}}^{\left(M\right)}\left(n\right)\right)=\frac{1}{1+exp(-\bar{\omega }{\tilde{X}}^{\left(M\right)}\left(n\right))}$$

The output of the logistic regression layer is a continuous probability rather than a hard binary value. For a discriminator input vector, the sigmoid output can be generally written as

(7) $$D\left(z\right)=\sigma \left(z\right)=\frac{1}{1+\exp(-z)}$$

During adversarial training, the measured ADOA vectors and generated ADOA vectors are assigned target labels 1 and 0 respectively, and the loss function is optimized directly using the continuous probability D(z). Therefore, no hard threshold is applied during back-propagation. The threshold is only used for reporting the discriminator decision or classification accuracy. Specifically, the decision rule is

(8) $$\hat{y}=\left\{\begin{array}{cc} 1, & D(z)\geq 0.5,\\ 0, & D(z)<0.5. \end{array}\right.$$

where the threshold 0.5 is used only for evaluation and does not participate in the gradient-based training process. It should be noted that the sigmoid activation may attenuate the gradient when the discriminator output approaches its asymptotic limits. The derivative of the sigmoid function is $\sigma^{\prime }\left(z\right)=\sigma \left(z\right)\left(1-\sigma (z)\right)$. Therefore, when the discriminator probability output approaches 0 or 1, the scaling factor becomes close to zero. In this saturated region, the discriminator classifies the input ADOA vectors with very high confidence, and the back-propagated gradient through the logistic regression layer and the auto-encoder layers is weakened. This attenuation may also reduce the gradient transferred to the geometric generator for updating the AP coordinates. Therefore, stable binary cross-entropy training and balanced discriminator–generator updates are used to avoid excessive discriminator saturation during adversarial training.

Due to

(9) $$\begin{array}{c} {\tilde{X}}^{\left(M\right)}\left(n\right)=F\left(\omega^{\left(M-1\right)}{\tilde{X}}^{\left(M-1\right)}\left(n\right)+b^{\left(M-1\right)}\right)\\ =F(\omega^{\left(M-1\right)}F\left(\cdots F\left(\omega^{\left(1\right)}{\tilde{X}}^{\left(1\right)}\left(n\right)+b^{\left(1\right)}\right)\cdots +b^{\left(M-2\right)}\right)\\ +b^{\left(M-1\right)}), \end{array}$$

the output of logistic regression network can be denoted as $D_{\bar{\omega },\omega^{\left(1\right)},b^{\left(1\right)},\cdots \omega^{\left(M\right)},b^{\left(M\right)}}\left({\tilde{X}}^{\left(1\right)}\left(n\right)\right)$.

By labeling the real AODA vector as 1 (true) and the generated vector as 0 (false), a supervised parameter estimation method can be employed to train the logistic regression network. Specifically, the objective function of the discriminator is constructed as follows:

(10) $$\begin{array}{c} J^{\prime }\left(\bar{\omega },\omega^{\left(1\right)},b^{\left(1\right)},\cdots \omega^{\left(M\right)},b^{\left(M\right)}\right)\\ =\frac{1}{N}\sum_{n=1}^{N}\{\log D_{\bar{\omega },\omega^{\left(1\right)},b^{\left(1\right)},\cdots \omega^{\left(M\right)},b^{\left(M\right)}}\left(\Xi \left(\psi \left(p_{n}\right)\right)\right)\\ +\log\left(1-D_{\bar{\omega },\omega^{\left(1\right)},b^{\left(1\right)},\cdots \omega^{\left(M\right)},b^{\left(M\right)}}\left(G_{a_{1},a_{2},\cdots ,a_{L}}\left({\bar{p}}_{n}\right)\right)\right)\} \end{array}$$

where $G_{a_{1},a_{2},\cdots ,a_{L}}\left({\bar{p}}_{n}\right)$ denotes the generated vector corresponding to random AP locations ${\bar{p}}_{n}$.

This objective function achieves its maximum when the discriminator outputs “1” and “0” for inputting a real ADOA vector and a generated ADOA vector, respectively, i.e., $D_{\bar{\omega },\omega^{\left(1\right)},b^{\left(1\right)},\cdots ,\omega^{\left(M\right)},b^{\left(M\right)}}(\Xi \left(\psi \left(p_{n}\right)\right)=1$ and $D_{\bar{\omega },\omega^{\left(1\right)},b^{\left(1\right)},\cdots \omega^{\left(M\right)},b^{\left(M\right)}}(G_{a_{1},a_{2},\cdots ,a_{L}}\left({\bar{p}}_{n}\right)=0$. Hence, the parameters of the logistic regression network can be updated by maximizing the above objective function with the classical gradient ascend method.

##### Fine-Tuning of Global Parameters

Considering that the label of the input data of the discriminator is known in a GAN, the classical back-propagation (BP) algorithm is employed to calculate the gradient of the objective function (7) with respect to the global parameters and fine-tune the global parameters. After the fine-tuning of the global parameters, the optimal discriminator can be obtained for a given generator. That is, the discriminator achieves its optimal ability of distinguishing the real AODA vectors from the generated ones.

#### 3.1.2. Training of the Generator

Considering that the real ADOA data possesses 2-dimensional characteristics, random 2-dimensional coordinates of the MT are chosen as the input of the generator. Correspondingly, the generator outputs the ‘fake’ ADOA vectors directly according to the geometric relationship among the AP coordinates, the MT position and the ADOA vector in (1), rather than through a deep neural network, which is the most straightforward model in traditional GANs. Hence the AP coordinates are the generator’s parameters to be optimized in our GAN.

The iterative topology-search process can be interpreted as follows. At each training iteration, a batch of random two-dimensional MT positions is sampled from the indoor coordinate region. Given the current AP-coordinate parameters, the explicit generator computes the corresponding generated ADOA vectors according to the geometric ADOA function. The discriminator then evaluates whether these generated ADOA vectors follow the same distribution as the measured ADOA vectors. The resulting adversarial loss is used to update the AP-coordinate parameters of the generator. Therefore, the topology search is not performed by adding or removing APs in a graphical procedure, but by continuously optimizing the AP-coordinate parameters in the explicit geometric generator. After convergence, the optimized AP-coordinate parameters are taken as the estimated AP topology.

Given the parameters and the input of the generator, that is, the coordinates of all the APs ${\left\{a_{l}\right\}}_{l=1}^{L}$ and the random MT coordinate ${\bar{p}}_{n}\in P$ with P denoting the 2-dimensional indoor random coordinate set, the generated ADOA ${\overset{˘}{\theta }}_{l_{1},l_{2}}\left({\bar{p}}_{n}\right)$ for the AP pair $\left\{AP_{l_{1}},AP_{l_{2}}\right\}$ at the random position ${\bar{p}}_{n}$ satisfies

(11) $$\cos{\overset{˘}{\theta }}_{l_{1},l_{2}}\left({\bar{p}}_{n}\right))=\frac{\left(a_{l_{1}}-{\bar{p}}_{n}\right)•\left(a_{l_{2}}-{\bar{p}}_{n}\right)}{\left|\left(a_{l_{1}}-{\bar{p}}_{n}\right)\right|\cdot \left|\left(a_{l_{2}}-{\bar{p}}_{n}\right)\right|}.$$

Thus, the generated ADOA vector at this random MT position can be expressed as

$$\begin{array}{c} G_{a_{1},a_{2},\cdots ,a_{L}}\left({\bar{p}}_{n}\right)\\ =[\cos\left({\overset{˘}{\theta }}_{1,2}\left({\bar{p}}_{n}\right)\right),\cos\left({\overset{˘}{\theta }}_{2,3}\left({\bar{p}}_{n}\right)\right),\cdots ,\cos\left({\overset{˘}{\theta }}_{L-1,L}\left({\bar{p}}_{n}\right)\right)] \end{array}$$

##### The Loss Function of the Generator

The generator of the proposed GAN aims to generate ADOA vectors with the same distribution characteristics as that of the real data. In other words, the generated data can not be differentiated from the real data by the discriminator. For this purpose, the generator is trained to minimize the following loss function with respect to the AP coordinates

(12) $$\begin{array}{c} L(a_{1},a_{2},\cdots ,a_{L})=\\ \frac{1}{N}\sum_{n=1}^{N}\left\{\log\left(1-D_{\bar{\omega },\omega^{\left(1\right)},b^{\left(1\right)},\cdots \omega^{\left(M\right)},b^{\left(M\right)}}\left(G_{a_{1},a_{2},\cdots ,a_{L}}\left({\bar{p}}_{n}\right)\right)\right)\right\}\\ s.t.\;\;\;\;\;a_{1}=\left[0,0\right],\;\;\;a_{2}=\left[0,1\right] \end{array}$$

wherein the constraint condition fixes the coordinates of $a_{1}$ and $a_{2}$ in order to resolve the AP coordinates’ ambiguity of rotation, translation and scaling. This loss function achieves its minimum as the discriminator outputs “1”s for each generated data $G_{a_{1},a_{2},\cdots ,a_{L}}\left({\bar{p}}_{n}\right)$, which means that the discriminator classifies the ‘fake’ data as the real one.

##### Optimizing the Generator’s Parameters

Based on the loss function (9), the gradient descent method is employed to iteratively optimize the generator’s parameters. Although the generation function is the explicit geometric relationship in (8), the error between the actual output and the ideal output of discriminator depends on not only the generation function but also the discriminator structure, which involves hidden layers. Hence, the BP algorithm is still needed to calculate the gradient of the errors with respect to the generator parameters.

Denote the error square between the actual output and the ideal output of the discriminator corresponding to the input of the *n*-th generated vector, $G_{a_{1},a_{2},\cdots ,a_{L}}\left({\bar{p}}_{n}\right)$, as

$$e_{n}={\left(1-D_{\bar{\omega },\omega^{\left(1\right)},b^{\left(1\right)},\cdots \omega^{\left(M\right)},b^{\left(M\right)}}\left(G_{a_{1},a_{2},\cdots ,a_{L}}\left({\bar{p}}_{n}\right)\right)\right)}^{2}$$

where “1” is the ideal output of the discriminator for inputting generated vectors. Denote the *i*-th element of the *n*-th generated vector as $G_{i}$, i.e., $G_{i}=\cos\left({\overset{˘}{\theta }}_{i,i+1}\left(p_{n}\right)\right)$.

Considering the BP-based gradient calculation is composed of *T* similar iterative steps, only the *t*-th step is exemplified as follows.

In the *t*-th iterative step, the coordinate of the *l*-th AP is updated as

(13) $${\hat{a}}_{l}\left(t\right)={\hat{a}}_{l}\left(t-1\right)-\alpha \frac{\partial e_{n}}{\partial a_{l}},\;\;\;l=3,\cdots ,L$$

where α denotes the learning rate.

The gradient of the error square with respect to $a_{l}$ is derived as

(14) $$\begin{array}{cc} \frac{\partial e_{n}}{\partial a_{l}} & =\sum_{i=1}^{L-1}\frac{\partial e_{n}}{\partial G_{i}}\frac{\partial G_{i}}{\partial a_{l}}\\ & =\frac{\partial e_{n}}{\partial G_{i=l-1}}\frac{\partial G_{i=l-1}}{\partial a_{l}}+\frac{\partial e_{n}}{\partial G_{i=l}}\frac{\partial G_{i=l}}{\partial a_{l}} \end{array}$$

where

$$\frac{\partial e_{n}}{\partial G_{i}}=\sum_{j=1}^{J^{\left(2\right)}}\frac{\partial e_{n}}{\partial z_{n,j}^{\left(1\right)}}\frac{\partial z_{n,j}^{\left(1\right)}}{\partial G_{i}}=\sum_{j=1}^{J^{\left(2\right)}}\left(-\delta_{n,k}^{\left(1\right)}\right)\left(w_{ij}^{\left(1\right)}\right)$$

where $z_{n,j}^{\left(1\right)}$ represents the output (before activation function) of the *j*-th neuron of the first layer of discriminator, $w_{ij}^{\left(1\right)}$ represents the corresponding weight of the first layer of the discriminator, $\delta_{n,j}^{\left(1\right)}$ denotes the BP gradient at the *j*-th neuron in the first layer of the discriminator, $J^{\left(2\right)}$ represents the number of neuron of the second layer and

(15) $$\begin{array}{c} \frac{\partial g_{i=l-1}}{\partial a_{l}}=\frac{\partial \frac{\left(a_{l-1}-{\bar{p}}_{n}\right)•\left(a_{l}-{\bar{p}}_{n}\right)}{\left|\left(a_{l-1}-{\bar{p}}_{n}\right)\right|\cdot \left|\left(a_{l}-{\bar{p}}_{n}\right)\right|}}{\partial a_{l}}\\ =\frac{\left(a_{l-1}-{\bar{p}}_{n}\right)}{\left|\left(a_{l-1}-{\bar{p}}_{n}\right)\right|\cdot \left|\left(a_{l}-{\bar{p}}_{n}\right)\right|}-\frac{\left(a_{l-1}-{\bar{p}}_{n}\right)•\left(a_{l}-{\bar{p}}_{n}\right)\left(a_{l}-{\bar{p}}_{n}\right)}{{\left(\left|\left(a_{l-1}-{\bar{p}}_{n}\right)\right|\cdot \left|\left(a_{l}-{\bar{p}}_{n}\right)\right|\right)}^{3}}\\ \frac{\partial g_{i=l}}{\partial a_{l}}=\frac{\partial \frac{\left(a_{l}-{\bar{p}}_{n}\right)•\left(a_{l+1}-{\bar{p}}_{n}\right)}{\left|\left(a_{l}-{\bar{p}}_{n}\right)\right|\cdot \left|\left(a_{l+1}-{\bar{p}}_{n}\right)\right|}}{\partial a_{l}}\\ =\frac{\left(a_{l+1}-{\bar{p}}_{n}\right)}{\left|\left(a_{l+1}-{\bar{p}}_{n}\right)\right|\cdot \left|\left(a_{l}-{\bar{p}}_{n}\right)\right|}-\frac{\left(a_{l+1}-{\bar{p}}_{n}\right)•\left(a_{l}-{\bar{p}}_{n}\right)\left(a_{l}-{\bar{p}}_{n}\right)}{{\left(\left|\left(a_{l+1}-{\bar{p}}_{n}\right)\right|\cdot \left|\left(a_{l}-{\bar{p}}_{n}\right)\right|\right)}^{3}} \end{array}$$

Define $\delta_{n,j}^{\left(m\right)}=-\frac{\partial e_{n}}{\partial z_{n,j}^{\left(m\right)}}$; then, the BP gradient, $\delta_{n,j}^{\left(1\right)}$, can be recursively obtained as

(16) $$\begin{array}{cc} \delta_{n,j}^{\left(m\right)}= & \sum_{i=1}^{J^{\left(m+1\right)}}\delta_{n,i}^{\left(m+1\right)}w_{ji}^{\left(m+1\right)},\\ & \;\;\;j=1,\cdots ,J^{\left(m\right)};m=2,\cdots ,M-1\\ \delta_{n,j}^{\left(M\right)} & =-2(1-z_{n,j}^{\left(M\right)}) \end{array}$$

Through iteratively training the discriminator and the generator, the proposed GAN system can generate ADOA vectors with the same distribution as the real vector finally. Consequently, the parameters of the generator converge to the optimal solution; that is, the optimal estimation of the AP position coordinates is obtained.

The proposed framework does not first generate a large number of candidate virtual APs and then eliminate false ones. The APs in the generator correspond to the dominant paths observed in the ADOA measurements, including the LOS path from the physical AP and the dominant single-reflection NLOS paths represented by virtual APs. Therefore, under the assumed dominant-path model, the GAN estimates the coordinates of these APs/virtual APs by matching the measured ADOA distribution, and false virtual AP elimination is not a separate step in the current framework. If spurious paths are included due to incorrect path detection or association, they may introduce outliers in the ADOA dataset and degrade the topology estimation accuracy; robust path selection or pruning strategies can be incorporated in future work.

The pseudocode for the proposed GAN-based AP topology estimation method is given in Algorithm 1.

### 3.2. Localization of the MT and Construction of the Environment Map

Based on the estimated AP positions above, the MT position for each ADOA vector can be further obtained. From the geometric relationship between the MT’s position and the APs’ positions in (1), the estimation of the MT position for each ADOA vector can be formulated as the following minimization problem:

(17) $$\begin{array}{c} {\hat{p}}_{n}=\arg\min_{p_{n}\in P}\sum_{{\overset{˘}{\theta }}_{l_{1},l_{2}}\left(p_{n}\right)\in \psi \left(p_{n}\right)}\{({\hat{a}}_{l_{1}}-p_{n})•\left({\hat{a}}_{l_{2}}-p_{n}\right)\\ {-\cos{\overset{˘}{\theta }}_{l_{1},l_{2}}\left(p_{n}\right)\left|{\hat{a}}_{l_{1}}-p_{n}\right|\cdot \left|{\hat{a}}_{l_{2}}-p_{n}\right|\}}^{2} \end{array}$$

Hence, each MT position can be obtained by searching discretely over possible indoor MT position-range P.
**Algorithm 1** GAN based AP topology estimation.**Input:** Observed ADOA vectors ${\left\{\tilde{\theta }\left(p_{n}\right)\right\}}_{n=1}^{N}$ at a large number of random and unknown positions
  1:Initialization: ${\hat{a}}_{3:L}^{0},{\hat{a}}_{1}=\left[0\;\;0\right],{\hat{a}}_{2}=\left[1\;\;0\right],t=0$  2:**for** number of training iterations **do**  3:    **Training the discriminator:**  4:    Sample minibatch of *N* observed ADOA vector samples from ${\left\{\tilde{\theta }\left(p_{n}\right)\right\}}_{n=1}^{N}$  5:    Train the auto-encoder neural network in the discriminator by descending its stochastic gradient with respect to the loss function $J(\omega^{\left(m\right)},b^{\left(m\right)},{\overset{´}{b}}^{\left(m\right)})$ in (4)    ${▿}_{\omega^{\left(m\right)},b^{\left(m\right)}}J\left(\omega^{\left(m\right)},b^{\left(m\right)},{\overset{´}{b}}^{\left(m\right)}\right)$  6:    Sample minibatch of *N* observed ADOA vector samples from ${\left\{\tilde{\theta }\left(p_{n}\right)\right\}}_{n=1}^{N}$  7:    Select *M* MT positions ${\left\{{\bar{p}}_{m}\right\}}_{m=1}^{M}$ randomly and generate ADOA vector samples Equation (8)  8:    Update the discriminator by ascending its stochastic gradient with respect to the objective function $J^{\prime }\left(\bar{\omega },\omega^{\left(1\right)},b^{\left(1\right)},\cdots \omega^{\left(M\right)},b^{\left(M\right)}\right)$ in (7)    ${▿}_{\omega^{\left(m\right)},b^{\left(m\right)}}J^{\prime }\left(\bar{\omega },\omega^{\left(1\right)},b^{\left(1\right)},\cdots \omega^{\left(M\right)},b^{\left(M\right)}\right)$  9:    **Training the generator:**10:    Select *M* MT positions ${\left\{{\bar{p}}_{m}\right\}}_{m=1}^{M}$ randomly and generate ADOA vector samples Equation (8)11:    Update the generator by descending its stochastic gradient with respect to the loss function $L(a_{1},a_{2},\cdots ,a_{L})$ in (9)    ${▿}_{a_{1},a_{2},\cdots ,a_{L}}L\left(a_{1},a_{2},\cdots ,a_{L}\right)$12:**end for**
**Output:** the AP position estimates


**Figure 4:**
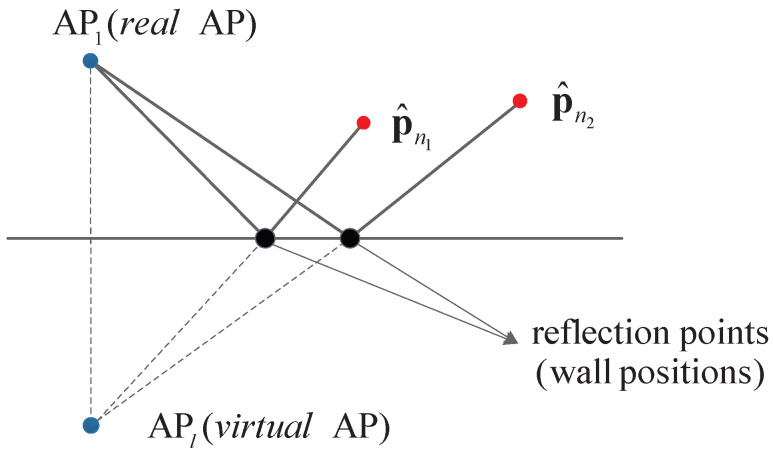
Illustration of the wall position estimation.

Finally, given by the estimated MT positions ${\left\{{\hat{p}}_{n}\right\}}_{n=1}^{N}$ and the estimated AP positions ${\left\{{\hat{a}}_{l}\right\}}_{l=1}^{L}$, the wall positions can be geometrically calculated as in [19], which are shown in Figure 4. For each estimated MT position $\left\{{\hat{p}}_{n}\right\}$ and each position pair $\left\{{\hat{a}}_{1},{\hat{a}}_{l}\right\}$ of the physical AP and a virtual AP, the reflection point on an indoor wall corresponding to a received NLOS path at $\left\{{\hat{p}}_{n}\right\}$ can be estimated as the intersection between the segment $\bar{{\hat{p}}_{n}{\hat{a}}_{l}}$ and the line that bisects segment $\bar{{\hat{a}}_{1}{\hat{a}}_{l}}$. Thus, the indoor walls, i.e., the environment map, can be constructed.

### 3.3. CRLB for the MT Localization

In this section, we derive the Fisher information matrix (FIM) and the Cramer–Rao lower bound (CRLB) of the estimation error of the MT position, and we compare the positioning accuracy of our algorithm with the CRLB of localization in Section 4.

From (1), the ADOA at position $p_{n}$ for the AP pair $\left\{AP_{l_{1}},AP_{l_{2}}\right\}$ can be derived as

$${\overset{˘}{\theta }}_{l_{1},l_{2}}\left(p_{n}\right)=\arccos\left\{\frac{\left(a_{l_{1}}-p_{n}\right)•\left(a_{l_{2}}-p_{n}\right)}{\left|\left(a_{l_{1}}-p_{n}\right)\right|•\left|\left(a_{l_{2}}-p_{n}\right)\right|}\right\}$$

Assume the random measurement error of the AOA follows zero-mean Gaussian distribution with variance of $\sigma^{2}$, thus the probability density function of the measured ADOA vector ψ at random and unknown MT positions ${\left\{p_{n}\right\}}_{n=1}^{N}$ is

(18) $$\begin{array}{c} P\left(\psi |\overset{˘}{\theta }\right)=\frac{1}{{\left(2\pi \right)}^{\frac{L-1}{2}}{\left(detC\right)}^{\frac{1}{2}}}\cdot \\ \exp\left\{-\frac{1}{2}{\left(\psi -\overset{˘}{\theta }\right)}^{T}C^{-1}\left(\psi -\overset{˘}{\theta }\right)\right\} \end{array}$$

where

$$\begin{array}{c} \psi ={\left[\psi {\left(p_{1}\right)}^{T},\psi {\left(p_{2}\right)}^{T},\cdots ,\psi {\left(p_{N}\right)}^{T}\right]}^{T}\\ \psi \left(p_{n}\right)={\left[{\hat{\theta }}_{1,2}\left(p_{n}\right),{\hat{\theta }}_{2,3}\left(p_{n}\right),\cdots ,{\hat{\theta }}_{L-1,L}\left(p_{n}\right)\right]}^{T}\\ \overset{˘}{\theta }={\left[\overset{˘}{\theta }{\left(p_{1}\right)}^{T},\overset{˘}{\theta }{\left(p_{2}\right)}^{T},\cdots ,\overset{˘}{\theta }{\left(p_{N}\right)}^{T}\right]}^{T}\\ \overset{˘}{\theta }{\left(p_{n}\right)}^{T}={\left[{\overset{˘}{\theta }}_{1,2}\left(p_{n}\right),\cdots ,{\overset{˘}{\theta }}_{L-1,L}\left(p_{n}\right)\right]}^{T} \end{array}$$

and C is the covariance matrix of the measured ADOA vector ψ. Since the measured ADOA vectors at different positions are independent from each other, the covariance matrix C is a block diagonal matrix as

(19) $$C=\left[\begin{array}{cccc} C^{\left(1\right)} & 0 & \cdots & 0\\ 0 & C^{\left(2\right)} & \cdots & 0\\ 0 & 0 & \ddots & 0\\ 0 & 0 & 0 & C^{\left(N\right)} \end{array}\right]$$

Moreover, it is not difficult to derive from ${\overset{˘}{\theta }}_{l_{1},l_{2}}\left(p_{n}\right)=\theta_{l_{1}}\left(p_{n}\right)-\theta_{l_{2}}\left(p_{n}\right)$ that the sub-covariance matrix $C^{\left(n\right)}$ satisfies

(20) $$C_{l_{1},l_{2}}^{\left(n\right)}=\left\{\begin{array}{c} 2\sigma^{2},\;\;if\;l_{1}=l_{2}\\ -\sigma^{2},\;if\;\left|l_{1}-l_{2}\right|=1\\ 0,\;\;\;\;\;else \end{array}\right.$$

Hence, the probability density function of the measured ADOA vector ψ can also be rewritten as

(21) $$\begin{array}{c} P\left(\psi |\overset{˘}{\theta }\right)=\frac{1}{{\left(2\pi \right)}^{\frac{L-1}{2}}{\left(detC\right)}^{\frac{1}{2}}}\cdot \\ \exp\left\{-\frac{1}{2}\sum_{n=1}^{N}{\left(\psi \left(p_{n}\right)-\overset{˘}{\theta }\left(p_{n}\right)\right)}^{T}{\left(C^{\left(n\right)}\right)}^{-1}\left(\psi \left(p_{n}\right)-\overset{˘}{\theta }\left(p_{n}\right)\right)\right\} \end{array}$$

#### 3.3.1. FIM of the ADOA Vector

The FIM of the ADOA vector ${\overset{˘}{\theta }}_{n}$ can be expressed as

(22) $$\begin{array}{c} J_{\overset{˘}{\theta }}=E_{P(\psi |\overset{˘}{\theta })}\left\{-\frac{\partial^{2}lnP(\psi |\overset{˘}{\theta })}{\partial \overset{˘}{\theta }\partial {\overset{˘}{\theta }}^{T}}\right\}. \end{array}$$

Due to the independence between the measured ADOA vectors at different positions, the FIM of the ADOA vector $\overset{˘}{\theta }$ is also a block diagonal matrix as

(23) $$J_{\overset{˘}{\theta }}=\left[\begin{array}{cccc} J_{\overset{˘}{\theta }\left(p_{1}\right)} & 0 & \cdots & 0\\ 0 & J_{\overset{˘}{\theta }\left(p_{2}\right)} & \cdots & 0\\ 0 & 0 & \ddots & 0\\ 0 & 0 & 0 & J_{\overset{˘}{\theta }\left(p_{N}\right)} \end{array}\right]$$

with

(24) $$\begin{array}{c} J_{\overset{˘}{\theta }\left(p_{n}\right)}=E_{P(\psi \left(p_{n}\right)|\overset{˘}{\theta }\left(p_{n}\right))}\left\{-\frac{\partial^{2}lnP(\psi \left(p_{n}\right)|\overset{˘}{\theta }\left(p_{n}\right))}{\partial \overset{˘}{\theta }\left(p_{n}\right)\partial {\overset{˘}{\theta }\left(p_{n}\right)}^{T}}\right\}\\ =-\frac{\partial^{2}\left\{-\frac{1}{2}{\left(\psi \left(p_{n}\right)-\overset{˘}{\theta }\left(p_{n}\right)\right)}^{T}{\left(C^{\left(n\right)}\right)}^{-1}\left(\psi \left(p_{n}\right)-\overset{˘}{\theta }\left(p_{n}\right)\right)\right\}}{\partial \overset{˘}{\theta }\left(p_{n}\right)\partial {\overset{˘}{\theta }\left(p_{n}\right)}^{T}}\\ =\frac{1}{2}\left({\left(C^{\left(n\right)}\right)}^{-1}+{\left\{{\left(C^{\left(n\right)}\right)}^{-1}\right\}}^{T}\right)={\left(C^{\left(n\right)}\right)}^{-1} \end{array}$$

#### 3.3.2. FIM of the MT Position and the AP Positions

Further, the FIM of the MT position and the AP positions is obtained through a transformation of variables from $\overset{˘}{\theta }$ to η, where $\eta ={\left[a_{3}^{T},\cdots ,a_{L}^{T},p_{1}^{T},\cdots ,p_{N}^{T}\right]}^{T}$.

(25) $$J_{\eta }=TJ_{\overset{˘}{\theta }}T^{T}$$

where the transformation matrix T is expressed as

(26) $$\begin{array}{c} T=\frac{\partial {\overset{˘}{\theta }}^{T}}{\partial \eta }\\ =\left[\begin{array}{ccc} \frac{\partial \overset{˘}{\theta }{\left(p_{1}\right)}^{T}}{\partial \eta (1)} & \cdots & \frac{\partial \overset{˘}{\theta }{\left(p_{N}\right)}^{T}}{\partial \eta (1)}\\ \frac{\partial \overset{˘}{\theta }{\left(p_{1}\right)}^{T}}{\partial \eta (2)} & \cdots & \frac{\partial \overset{˘}{\theta }{\left(p_{N}\right)}^{T}}{\partial \eta (2)}\\ \vdots & \cdots & \vdots \\ \frac{\partial \overset{˘}{\theta }{\left(p_{1}\right)}^{T}}{\partial \eta (L-2+N)} & \cdots & \frac{\partial \overset{˘}{\theta }{\left(p_{N}\right)}^{T}}{\partial \eta (L-2+N)} \end{array}\right] \end{array}$$

with

(27) $$\begin{array}{c} \frac{\partial \overset{˘}{\theta }{\left(p_{n}\right)}^{T}}{\partial \eta (m)}=\left[\begin{array}{ccc} \frac{\partial {\overset{˘}{\theta }}_{1,2}\left(p_{n}\right)}{\partial \eta (m)} & \cdots & \frac{\partial {\overset{˘}{\theta }}_{L-1,L}\left(p_{n}\right)}{\partial \eta (m)} \end{array}\right] \end{array}$$

and

(28) $$\begin{array}{c} \frac{\partial {\overset{˘}{\theta }}_{l,l+1}\left(p_{n}\right)}{\partial \eta (m)}=\frac{\partial {\overset{˘}{\theta }}_{l,l+1}\left(p_{n}\right)}{\partial a_{\left(m+2\right)}},\;m\leq L-2\\ =\left\{\begin{array}{c} -\frac{1}{\|p_{n}-a_{l}{\|}_{2}}{\left[sin\left(\theta_{l}\left(p_{n}\right)\right),-cos\left(\theta_{l}\left(p_{n}\right)\right)\right]}^{T},\\ \;\;\;\;\;\;\;\;\;\;if\;(m+2)=l\\ \frac{1}{\|p_{n}-a_{\left(l+1\right)}{\|}_{2}}{\left[sin\left(\theta_{l+1}\left(p_{n}\right)\right),-cos\left(\theta_{l+1}\left(p_{n}\right)\right)\right]}^{T},\\ \;\;\;\;\;\;\;\;\;\;if\;(m+2)=l+1\\ 0,\;\;\;\;\;else \end{array}\right. \end{array}$$

(29) $$\begin{array}{c} \frac{\partial {\overset{˘}{\theta }}_{l,l+1}\left(p_{n}\right)}{\partial \eta (m)}=\frac{\partial {\overset{˘}{\theta }}_{l,l+1}\left(p_{n}\right)}{\partial p_{\left(m-L+2\right)}},\;\;m>L-2\\ =\left\{\begin{array}{c} \frac{1}{\|p_{n}-a_{l}{\|}_{2}}{\left[sin\left(\theta_{l}\left(p_{n}\right)\right),-cos\left(\theta_{l}\left(p_{n}\right)\right)\right]}^{T}-\\ \frac{1}{\|p_{n}-a_{\left(l+1\right)}{\|}_{2}}{\left[sin\left(\theta_{l+1}\left(p_{n}\right)\right),-cos\left(\theta_{l+1}\left(p_{n}\right)\right)\right]}^{T},\\ \;\;\;\;\;\;\;\;\;\;if\;(m-L+2)=n\\ 0,\;\;\;\;\;else \end{array}\right. \end{array}$$

It can be derived by substituting (23) into (22) that the FIM $J_{\eta }$ satisfies

(30) $$J_{\eta }\left(2r-1:2r,2s-1:2s\right)=\sum_{n=1}^{N}\frac{\partial \overset{˘}{\theta }{\left(p_{n}\right)}^{T}}{\partial \eta (r)}J_{\overset{˘}{\theta }\left(p_{n}\right)}\frac{\partial \overset{˘}{\theta }\left(p_{n}\right)}{\partial \eta (s)}$$

By integrating (25) and (26) into (27), the FIM $J_{\eta }$ can be partitioned as

(31) $$\begin{array}{c} J_{\eta }=\left[\begin{array}{c} \begin{array}{cc} A_{2(L-2)\times 2(L-2)} & B_{2(L-2)\times (2N)}\\ C_{2N\times 2(L-2)} & D_{2N\times 2N} \end{array} \end{array}\right] \end{array}$$

with

(32) $$\begin{array}{c} A(2r-1:2r,2s-1:2s)\\ =\sum_{n=1}^{N}\frac{\partial \overset{˘}{\theta }{\left(p_{n}\right)}^{T}}{\partial \eta (r)}J_{\overset{˘}{\theta }\left(p_{n}\right)}\frac{\partial \overset{˘}{\theta }\left(p_{n}\right)}{\partial \eta (s)}=\sum_{n=1}^{N}\frac{\partial \overset{˘}{\theta }{\left(p_{n}\right)}^{T}}{\partial a_{r+2}}J_{\overset{˘}{\theta }\left(p_{n}\right)}\frac{\partial \overset{˘}{\theta }\left(p_{n}\right)}{\partial a_{s+2}}\\ \;\;\;\;\;\;\;\;\;\;\;\;r=1,2,\cdots ,L-2;s=1,2,\cdots ,L-2\\ B(2r-1:2r,2s-1:2s)\\ =\frac{\partial \overset{˘}{\theta }{\left(p_{s}\right)}^{T}}{\partial \eta (r)}J_{\overset{˘}{\theta }\left(p_{s}\right)}\frac{\partial \overset{˘}{\theta }\left(p_{s}\right)}{\partial \eta (s+L-2)}=\frac{\partial \overset{˘}{\theta }{\left(p_{s}\right)}^{T}}{\partial a_{r+2}}J_{\overset{˘}{\theta }\left(p_{s}\right)}\frac{\partial \overset{˘}{\theta }\left(p_{s}\right)}{\partial p_{s}}\\ \;\;\;\;\;\;\;\;\;\;\;\;\;\;r=1,2,\cdots ,L-2;s=1,2,\cdots ,N\\ C(2r-1:2r,2s-1:2s)\\ =\frac{\partial \overset{˘}{\theta }{\left(p_{r}\right)}^{T}}{\partial \eta (r+L-2)}J_{{\overset{˘}{\theta }}_{r}}\frac{\partial \overset{˘}{\theta }\left(p_{r}\right)}{\partial \eta (s)}=\frac{\partial \overset{˘}{\theta }{\left(p_{r}\right)}^{T}}{\partial p_{r}}J_{\overset{˘}{\theta }\left(p_{r}\right)}\frac{\partial \overset{˘}{\theta }\left(p_{r}\right)}{\partial a_{s+2}}\\ \;\;\;\;\;\;\;\;\;\;\;\;\;\;r=1,2,\cdots ,N;s=1,2,\cdots ,L-2\\ D(2r-1:2r,2s-1:2s)\\ =\delta \left(r-s\right)\frac{\partial \overset{˘}{\theta }{\left(p_{r}\right)}^{T}}{\partial \eta (r+L-2)}J_{\overset{˘}{\theta }\left(p_{r}\right)}\frac{\partial \overset{˘}{\theta }\left(p_{s}\right)}{\partial \eta (s+L-2)}\\ =\delta \left(r-s\right)\frac{\partial \overset{˘}{\theta }{\left(p_{r}\right)}^{T}}{\partial p_{r}}J_{\overset{˘}{\theta }\left(p_{r}\right)}\frac{\partial \overset{˘}{\theta }\left(p_{s}\right)}{\partial p_{s}}\\ \;\;\;\;\;\;\;\;\;\;\;\;\;\;\;\;r=1,2,\cdots ,N;s=1,2,\cdots ,N \end{array}$$

It is worth mentioning that the submatrix D is a block diagonal matrix and each block is a 2×2 matrix.

According to the inversion property of partitioned matrices, the inverse of the FIM $J_{\eta }$ can be expressed as

(33) $$\begin{array}{c} J_{\eta }^{-1}=\\ \left[\begin{array}{c} \begin{array}{c} \begin{array}{cc} {\left(A-BD^{-1}C\right)}^{-1} & -{\left(A-BD^{-1}C\right)}^{-1}BD^{-1}\\ -D^{-1}C{\left(A-BD^{-1}C\right)}^{-1} & D^{-1}+D^{-1}C{\left(A-BD^{-1}C\right)}^{-1}BD^{-1} \end{array} \end{array} \end{array}\right] \end{array}$$

Considering that the submatrix D is a block diagonal matrix, its inverse $D^{-1}$ is also a block diagonal matrix and each block is the inverse of the corresponding block in the submatrix D. Moreover, the dimension of ${\left(A-BD^{-1}C\right)}^{-1}$ is 2(L−2)×2(L−2). As a result, the inversion of the FIM $J_{\eta }$ does not involve high computation complexity from (30).

#### 3.3.3. Bounds on Errors of the MT Position and the AP Positions

The mean squared error (MSE) of $\eta ={\left[a_{3},\cdots ,a_{L},p_{1},\cdots ,p_{N}\right]}^{T}$ is bounded as [27]

$$E_{P(\psi \left(p_{n}\right)|{\overset{˘}{\theta }}_{n})}\left[\left(\hat{\eta }-\eta \right){\left(\eta -\eta \right)}^{T}\right]\geq J_{\eta }^{-1}$$

Therefore, the position error bounds (PEBs) of the AP and the MT are respectively obtained as

(34) $$\begin{array}{c} {PEB}_{a_{l}}=\sqrt{tr\{{\left[J_{\eta }^{-1}\right]}_{\left(2l-5):(2l-4),(2l-5):(2l-4\right)}\}},\\ l=3,4,\cdots \cdots ,L \end{array}$$

(35) $$\begin{array}{c} {PEB}_{p_{n}}=\sqrt{tr\{{\left[J_{\eta }^{-1}\right]}_{2n^{\prime }-1:2n^{\prime },2n^{\prime }-1:2n^{\prime }}\}},\\ \;\;\;\;\;\;\;\;\;\;\;\;n^{\prime }=\left(L-2+n\right),\;n=1,2,\cdots \cdots ,N \end{array}$$

## 4. Simulation Results

**Figure 5:**
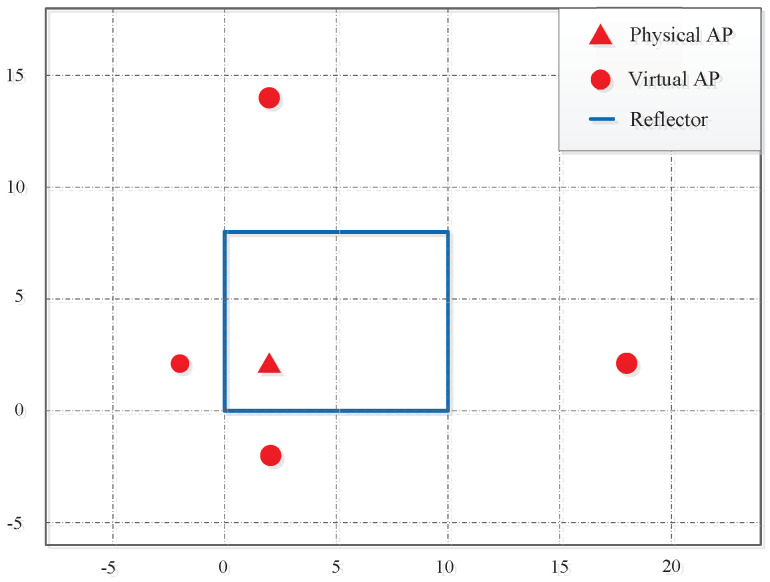
The 2-dimension layout of the indoor mmW WLAN environment.

In order to evaluate the performance of the proposed algorithm, we have conducted computer simulations in a relatively simple 10m×8m(length×width) 2-dimension indoor WLAN environment, in which there is only one physical AP located at (0.5,4.5,2.5) and four reflective walls. The 2-dimension layout of the indoor WLAN environment is shown in Figure 5. The classical ray-tracing propagation model [10] is adopted in the simulations to generate the multipath AOAs at random indoor positions. Although more recent ray-tracing tools, such as Sionna RT and MATLAB 2022a ray-tracing functions with STL indoor models [28], can generate more realistic channel responses by considering material properties, blockage, diffuse scattering, and antenna patterns, the simulations in this work mainly focus on the multipath geometric relationship among the physical/virtual APs, the MT positions, and the ADOA vectors. And the AOA measurement error is modeled as a zero-mean Gaussian random error [9] to represent the practical uncertainty caused by beam training, channel estimation, and AOA extraction. Therefore, no specific antenna-array configuration or fixed number of antenna elements is explicitly adopted in the simulation. In the simulations, the mmWave propagation model is simplified according to its severe path loss and quasi-optical propagation characteristics in air [10]. Specifically, only the LOS component and the first-order specular reflections from the side walls are retained, while higher-order reflected paths are neglected. Accordingly, the four virtual APs are generated as the mirror images of the physical AP with respect to the four corresponding reflecting walls.

Similar to the proposed algorithm, the JADE algorithm in [9] can also estimate the AP positions and the MT positions jointly by employing the geometric relationship between the APs and the MT based on the multipath ADOA measurements in indoor environments with only one AP deployed. Hence, we compare their localization and mapping performance in the simulations. Although many SLAM or JLAM methods have been proposed in the literature [20,29,30], they usually require additional information beyond what is directly available from the mmWave communication system, such as known floorplans, known reflector positions, synchronization information, AOD measurements, array orientation, or other prior environmental knowledge. In contrast, the proposed GAN-based JLAM method works under a zero-initial-information setting using only the collected ADOA measurements, and therefore the JADE algorithm is selected as the main baseline because it follows comparable information assumptions and can jointly estimate the AP topology and MT positions without prior floorplan information. In addition, the Cramer–Rao lower bound of localization is simulated as the ideal performance benchmark for mmW positioning. In the proposed GAN system, the discriminator is modeled as a three-layer auto-encoder neural network with 100 neurons in each layer.

The present simulation considers one physical AP and four virtual APs generated by single-bounce reflections, which is a typical virtual-AP-based mmWave SLAM scenario and is consistent with the comparison setting of the JADE algorithm. When multiple physical APs and/or more virtual APs are adopted, the ADOA dataset will contain more spatial angle observations and more independent geometric constraints. Therefore, the learned ADOA manifold becomes richer, and the AP topology and MT position estimation ambiguity can be further reduced. As a result, the cumulative distribution curve of estimation errors is expected to shift toward smaller error values compared with the single-physical-AP case. However, multiple physical APs and/or more virtual APs also increase the ADOA vector dimension, the number of AP topology parameters, the neural network training overhead, and the complexity of multipath data association.

### 4.1. Computational Complexity Comparison

The computational cost of the proposed GAN-based JLAM method mainly comes from the auto-encoder-based discriminator training and the geometric generator update. For one discriminator update, the forward and backward propagation complexity is approximately $O\left(\sum_{r=1}^{R}d_{r-1}d_{r}\right)$, where *N* is the number of measured ADOA vectors and $d_{r-1}\times d_{r}$ is the weight matrix size of the *r*-th neural layer. Since the generator is constrained by the explicit ADOA geometric model, one generator update mainly requires the calculation of generated ADOA vectors and their gradients with respect to AP coordinates, with complexity approximately zO(NL).

Therefore, the total complexity of the proposed adversarial training can be expressed as $O\left(T_{\mathrm{GAN}}\left[K_{D}N\sum_{r=1}^{R}d_{r-1}d_{r}+K_{G}NL\right]\right)$ where $T_{\mathrm{GAN}}$ is the number of adversarial iterations, and $K_{D}$ and $K_{G}$ are the numbers of discriminator and generator updates in each iteration. In comparison, the JADE algorithm mainly relies on iterative least-squares estimations, and its complexity can be written as $O\left(L^{2}\left(NT_{\mathrm{JADE}}+G\right)\right)$. The proposed method therefore introduces additional neural network training overhead compared with the least-squares iterations in JADE. However, the proposed generator is an explicit geometric generator rather than a fully trainable neural network, which reduces the number of trainable parameters. Due to the limited revision time, we did not add a new execution-time simulation, but the above complexity comparison clarifies the additional overhead and the computational difference between the proposed method and JADE.

### 4.2. Software Implementation

The proposed GAN-based JLAM algorithm was implemented using Python 3.11 numerical routines. In contrast to a conventional GAN with a black-box neural network generator, the generator in the proposed framework is constructed as an explicit geometric ADOA mapping function. The trainable parameters of the generator are the coordinates of the candidate physical and virtual APs, and the generated samples are calculated directly from the geometric relationship among the AP coordinates, the random MT coordinates, and the ADOA measurements. The discriminator is implemented as a deep auto-encoder-based neural network followed by a logistic regression layer. In the simulations, the discriminator is modeled as a three-layer auto-encoder neural network with 100 neurons in each layer.

The complete implementation is currently maintained as internal research code and is not publicly released at this stage, because it contains unpublished extensions and project-specific modules that are not directly related to the core algorithm presented in this paper. Nevertheless, all essential algorithmic components required for reproducing the reported results are described in the manuscript, including the explicit generator function, the discriminator structure, the loss functions, the training procedure, the initialization setting, and the simulation parameters.

### 4.3. Comparison of Localization Performance

**Figure 6:**
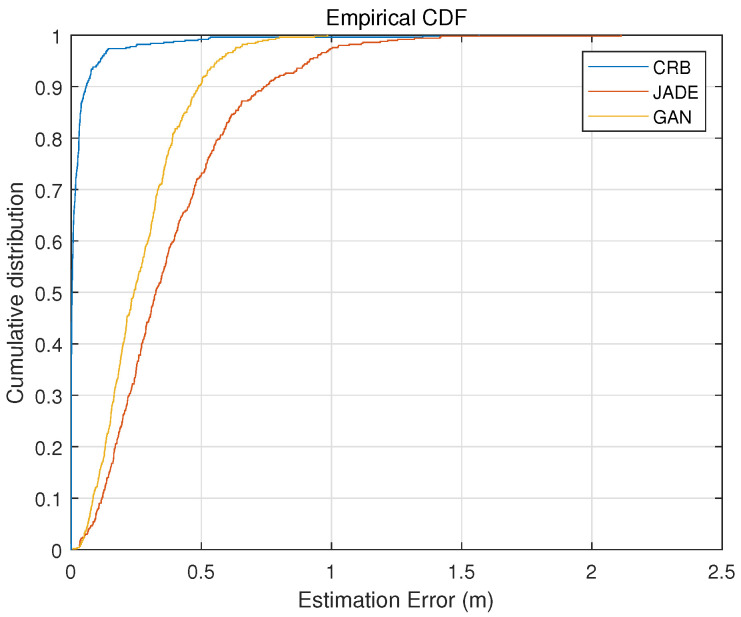
The cumulative probability distribution curves of localization error.

To show the localization performance of the proposed algorithm, the JADE algorithm and the Cramer–Rao lower bound, their cumulative probability distribution curves of errors in positioning the MT are presented in Figure 6. In this comparison, N=2000 ADOA vectors are collected at random indoor MT positions, and the standard deviation of the AOA measurement noise is set to 2°. The JADE method addresses the otherwise NP-hard joint AP-and-MT estimation problem by converting it into an iterative least-squares framework, where the AP coordinates and the MT positions are alternately updated through two LS estimation steps. Although this strategy reduces the optimization difficulty, it relies on a relaxation of the original geometric constraints linking the AP positions, MT positions, and ADOA observations. As a result, some useful geometric information contained in the measured ADOA data may not be fully preserved. On the other hand, the proposed algorithm uses a large number of ADOA vector measurements and the geometric relationship to train the generative adversarial network without any relaxation of the geometric constraint, thus can extract more information from the measured ADOA data than the JADE algorithm. This explains why the proposed algorithm performs better than the JADE algorithm in Figure 6.

Furthermore, Figure 6 indicates that the proposed GAN-based JLAM method has not yet reached the ideal Cramer–Rao lower bound, and a visible performance gap remains between their cumulative error distributions. This is because the proposed GAN-based algorithm is subject to insufficient ADOA vector measurements and the non-optimal selection of the number of neural layers and neurons in the discriminator. Moreover, the final MT positioning stage is performed over a discretized two-dimensional spatial grid, which also introduces a quantization error floor. Even if the adversarial network estimates the physical and virtual AP topology accurately, the estimated MT position is restricted to one of the predefined grid points. Therefore, the discretization step size limits the minimum achievable positioning error. It should be noted that the Cramer–Rao lower bound is not a practical localization algorithm but an ideal theoretical lower bound derived under the assumed noise model and unbiased-estimator conditions. Therefore, practical methods such as the proposed GAN-based JLAM method and JADE are expected to perform worse than the Cramer–Rao lower bound due to finite ADOA samples, adversarial training approximation, AP topology estimation errors, discretized position search, and measurement noise.

**Figure 7:**
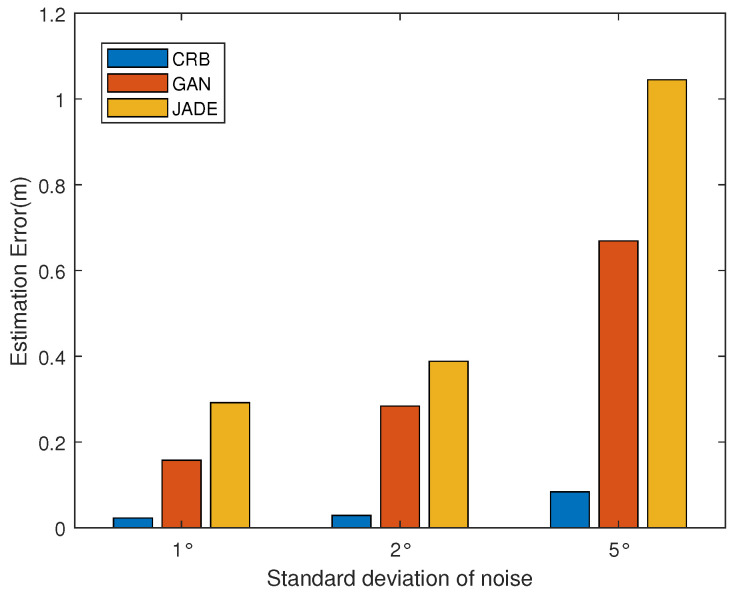
Localization performance versus AOA measurement noise.

Figure 7 presents the average MT localization error of the proposed GAN-based JLAM method, the JADE algorithm, and the Cramer–Rao lower bound under different ADOA noise levels. In this set of simulations, the number of measured ADOA vectors collected from random indoor positions is fixed at N=2000. As shown in Figure 7, the positioning accuracy of both the proposed method and JADE improves as the standard deviation of the ADOA measurement noise decreases, since more accurate angular observations provide more reliable geometric constraints and reduce the averaged influence of measurement errors. Moreover, the proposed method consistently achieves a much smaller localization error than JADE. This improvement is mainly attributed to the fact that the proposed GAN-based framework can exploit the geometric information embedded in the ADOA dataset more effectively and can maintain better robustness under noisy measurements. By contrast, JADE relies on LS-based estimation steps, whose performance is more easily affected by measurement noise.

**Figure 8:**
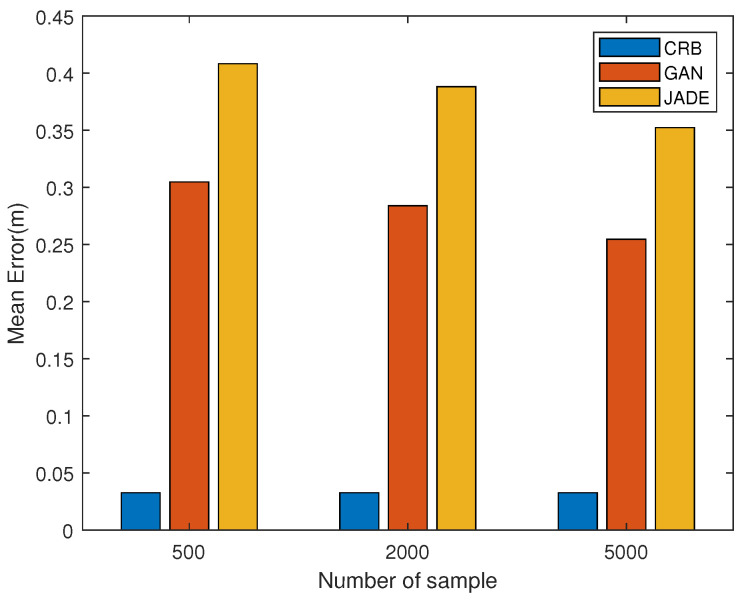
Localization performance versus the number of measured ADOA vectors.

Figure 8 shows the localization performance of the proposed algorithm and the JADE algorithm against the number of ADOA vector measurements. The standard deviation of Gaussian noise is set to 2°. It can be seen that the localization error of both the proposed algorithm and the JADE algorithm decreases with the increase in the number of samples due to the averaging effects. In addition, the proposed algorithm performs better than the JADE algorithm mainly due to the information loss caused by the relaxation of the geometric constraint in the JADE algorithm.

### 4.4. Comparison of Mapping Performance

**Figure 9:**
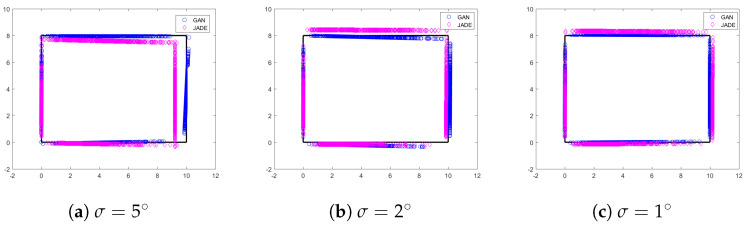
The mappingperformance for different standard deviations of ADOA errors: N=2000.

**Figure 10:**
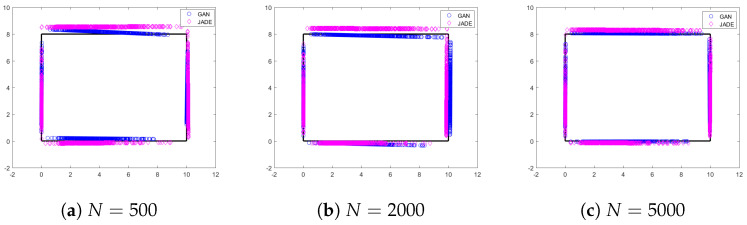
The mappingperformance for different numbers of ADOA vectors: σ=2°.

We now test the room boundary estimation capabilities, i.e., the mapping performance of the proposed method as explained in Section 4.4. Figure 9 and Figure 10 demonstrate the mapping performances of the proposed algorithm and the JADE algorithm versus the ADOA measurement noise and the number of ADOA vector measurements, respectively. The number of measured ADOA vectors at random indoor positions is set to 2000 in Figure 9 and the standard deviation of Gaussian noise is set to 2° in Figure 10. Similarly, Figure 9 and Figure 10 show that both the proposed algorithm and the JADE algorithm achieve better mapping performance with the standard deviation of the ADOA measurement noise decreasing or with the increase in the number of samples. Moreover, it is obvious that the proposed algorithm estimates the room boundary more accurately than the JADE algorithm in all cases in Figure 9 and Figure 10, because the proposed method requires no relaxation of the geometric constraint and thus achieves higher utilization of information contained in the ADOA measurement data. This further confirms the merit of the proposed GAN-based SLAM algorithm.

## 5. Conclusions

In this paper, we proposed a novel generative adversarial network (GAN)-based joint localization and mapping (JLAM) method for millimeter-wave (mmWave) communication systems. The proposed method exploits the two-dimensional distribution characteristics of high-dimensional angle difference of arrival (ADOA) vectors collected at a large number of random and unknown mobile terminal (MT) positions. Different from conventional GAN structures, the generator in the proposed framework is constructed as an explicit geometric ADOA function, whose trainable parameters are the physical and virtual access point (AP) coordinates. Therefore, the proposed method can learn the distribution of unlabeled ADOA measurements and transform them into the AP geometric topology. Based on the estimated AP topology, the MT positions can be further estimated and the indoor map can be constructed without requiring any a priori knowledge of the indoor environment.

The simulation results quantitatively confirm the effectiveness of the proposed method. Under the representative setting with N=2000 measured ADOA vectors and AOA measurement noise standard deviation σ=2°, the proposed method achieves an average localization error of about 0.25 m, while the JADE algorithm achieves about 0.60 m, corresponding to an error reduction of approximately 58%. The mapping results also show that the proposed method estimates the room boundary more accurately than JADE under different noise levels and different numbers of ADOA measurements. These results demonstrate that the proposed GAN-based JLAM method can efficiently exploit unlabeled ADOA measurements and achieve improved localization and mapping performance for mmWave communication systems.

## Data Availability

The original data presented in the study are openly available in the Supplementary Materials: Simulation Results for GAN-based JLAM.zip.

## Supplementary Materials

The following supporting information can be downloaded at https://www.mdpi.com/article/10.3390/s26134319/s1.

## Abbreviations

The following abbreviations are used in this manuscript:

SLAM	Simultaneous Localization and Mapping
GAN	Generative Adversarial Networks
mmW	Millimeter Wave
AP	Access Point
ADOA	Angle Difference of Arrival
MT	Mobile Terminal
MPCs	Multipath Components
JADE	Joint Anchor and Device location Estimation
LS	Least-Squares
JLAM	Joint Localization and Mapping
CLAM	Communication-driven Localization and Mapping
NLOS	Non-Line of Sight
2D/3D	2-dimension/3-dimension
