# Supplementary figures and images for: Generative Adversarial Network-Based Joint Mapping and Localization for Millimeter-Wave Communication Systems
Source: Sensors (Basel). 2026 Jul 7;26(13):4319. doi: 10.3390/s26134319 (PMC13364231; doi:10.3390/s26134319)

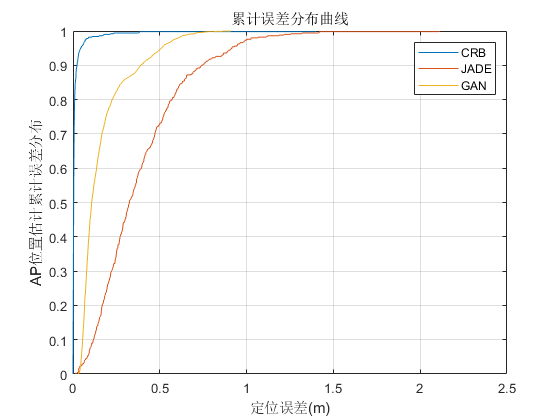

Supplement: Supplementary file 1 [file sensors-26-04319-s001.zip › noise2_cdf.png]
